# Large variation in mitochondrial DNA of sexual and parthenogenetic *Dahlica triquetrella* (Lepidoptera: Psychidae) shows multiple origins of parthenogenesis

**DOI:** 10.1186/1471-2148-13-90

**Published:** 2013-04-26

**Authors:** Jelmer A Elzinga, Jukka Jokela, Lisa NS Shama

**Affiliations:** 1 Department of Environmental and Biological Sciences, University of Jyväskylä, P.O. Box 35, 40014, Jyväskylä, Finland; 2 EAWAG, Swiss Federal Institute of Aquatic Science and Technology, and ETH-Zürich, Institute of Integrative Biology, Ueberlandstrasse 133, 8600, Dübendorf, Switzerland; 3 Current address: Alfred Wegener Institute, Helmholtz Centre for Polar and Marine Research, Wadden Sea Station Sylt, Hafenstraße 43, D-25992, List/Sylt, Germany

**Keywords:** Asexual, Automixis, COI, COII, Phylogeny, Tetraploid

## Abstract

**Background:**

Obligate parthenogenesis is relatively rare in animals. Still, in some groups it is quite common and has evolved and persisted multiple times. These groups may provide important clues to help solve the ‘paradox of sex’. Several species in the Psychidae (Lepidoptera) have obligate parthenogenesis. *Dahlica triquetrella* is one of those species where multiple transitions to parthenogenesis are postulated based on intensive cytological and behavioural studies. This has led to the hypothesis that multiple transitions from sexuals to diploid parthenogens occurred during and after the last glacial period, followed by transitions from parthenogenetic diploids to parthenogenetic tetraploids. Our study is the first to test these hypotheses using a molecular phylogeny based on mtDNA from multiple sexual and parthenogenetic populations from a wide geographic range.

**Results:**

Parthenogenetic (and sexual) *D. triquetrella* are not monophyletic, and considerable sequence variation is present suggesting multiple transitions to parthenogenesis. However, we could not establish ancestral sexual haplotypes from our dataset. Our data suggest that some parthenogenetic clades have evolved, indicating origins of parthenogenesis before the last glacial period.

**Conclusions:**

Multiple transitions to parthenogenesis have taken place in *Dahlica triquetrella*, confirming previous hypotheses. The number of different parthenogenetic clades, haplotypes and their apparent evolutionary age, clearly show that parthenogenesis has been a very successful reproductive strategy in this species over a long period.

## Background

Less than 1% of animal and plant species show some form of parthenogenetic reproduction, where offspring are produced via embryos without fertilisation [1-3]. Obligate parthenogenesis is often considered ‘an evolutionary dead end’ due to the reduction of genetic recombination [4,5]. However, parthenogenetic organisms avoid the ‘two-fold cost of sex’, potentially doubling the effective reproductive rate due to the absence of male progeny [4]. Furthermore, they may avoid costs associated with mating behaviour e.g. predation risks while searching for a partner or contracting a sexually transmitted disease [6]. Parthenogenetic organisms also do not face the risk of remaining unmated, assuring reproduction in sparse populations and newly invaded habitats for species with low mobility [7-9].

Obligate parthenogenetic reproduction has evolved at least a few times in most classes of higher organisms [1,3]. Three main evolutionary routes from sexual to obligate parthenogenetic reproduction can be distinguished. First, an intra- or inter-specific hybridisation event can lead to a parthenogenetic species or form. Often this is associated with polyploidisation. For example, almost all parthenogenetically reproducing (apomictic) plant species are polyploids and are believed to have originated through hybridization [10]. Many, if not most, parthenogenetic animal species are also believed to have originated from hybridization events, although polyploidy is not always involved [3,11]. A second pathway is the transition to a parthenogenetic form from a sexual without hybridisation. Several diploid, parthenogenetic animals show no evidence for hybridization (for example Timema stick insects [12]). Finally, parthenogenesis is frequently induced by *Wolbachia* bacteria [13], for instance in arthropod species with arrhenotokous development (where males develop from unfertilized eggs). Irrespective of the mechanism, the appearance and persistence of a parthenogenetic form seems to be extremely rare for a species. In some groups, however, parthenogenesis has occurred quite frequently [11]. Well-known examples are, for instance, oribatid mites, phasmids and weevils where several independent origins of parthenogenesis have been shown using phylogenetic analyses [3,11,14,15]. Reasons for the apparent success of parthenogenesis in these groups of organisms remain speculative, but such parthenogenetic groups may provide important clues to help explain the ‘paradox of sex’.

Although rare in Lepidoptera, parthenogenesis is known from at least five genera in four subfamilies of Psychidae [16]. In the Naryciinae, three species of parthenogens are known, among which is *Dahlica triquetrella* (Hubner, 1813). Interestingly, *D. triquetrella* diploid sexuals, diploid parthenogens and tetraploid parthenogens exist [17]. Based mainly on rearing experiments and cytogenetic observations, it is believed that parthenogenetic reproduction has evolved and persisted independently multiple times in this species (see e.g. [1,17]). Seiler [17] proposed that the evolution of parthenogenesis in *D. triquetrella* occurred in two steps. First, the production of (diploid parthenogenetic) offspring without copulation could have been achieved through the rare production of offspring from unfertilised eggs, which has been observed in many other insect species [8,18]. Indeed, virgin (sexual) *D. triquetrella* females occasionally oviposit, although viable offspring have never been observed [19]. However, this diploid parthenogenesis is considered relatively unstable in *D. triquetrella*, with eggs often failing to develop, and reproduction with sexuals still easily occurring [20]. The suggested next step is that tetraploid parthenogens (with a more stable parthenogenesis) evolved from a diploid parthenogenetic population via autopolyploidisation.

Importantly, it was suggested that the transition from diploid sexuals to (eventually tetraploid) parthenogens has occurred relatively frequently and may continue to do so. Evidence for this includes the fact that the initial cytological processes involved in the central fusion (the formation of the so-called ‘Richtungs-Kopulations-Kern)’ also occur in sexuals [21]. The existence of two forms of diploid sexuals and parthenogens, with a Z/ZZ female/male sex-determination system and those with a ZW/ZZ sex-determination system (where the ‘W’ chromosome is supposedly ‘empty’), also suggests at least two independent transitions to parthenogenesis [19,22,23]. Finally, Seiler [21] found that different populations of diploid parthenogens (and also tetraploids) vary in their abilities to fertilise their eggs after copulation, suggesting that a transition to parthenogenesis happened at different times in the past for these lines.

Seiler [17] also proposed a rough estimate of the time of divergence, based on the geographical distribution of the different reproductive types. He suggested that diploid sexuals survived the last glacial period (Würm) in ice-free refugia just north of the Alps, where they can still be found today [17]. When the ice retreated about 20 000 years ago, it likely left many areas sparsely populated allowing recently formed diploid parthenogens to outcompete sexuals and spread north and southwards. Later, tetraploids (which are slightly larger and have higher reproductive rates than diploid parthenogens) outcompeted diploid parthenogens in terms of dispersal [24], and spread to a wide area outside the Alps. Alternatively diploid parthenogens were outcompeted in large areas after their dispersal [22].

When Seiler studied *D. triquetrella* more than 60 years ago, his hypothesis of multiple independent origins of parthenogenesis could not be tested with phylogenetics. This was first conducted in the 1970’s with Swiss and Finnish populations using allozymes to test the hypothesis of multiple origins [22]. Here, large allozyme variation was found between parthenogenetic populations, especially within Switzerland, suggesting that monophyly was unlikely. However, a phylogenetic analysis using this data was not done at that time because of difficulties in establishing the number of alleles for polyploids. A later study produced a phylogeny for these data using a method where the number of alleles need not be known [25]. Again, polyphyly was suggested but the authors could not convincingly separate the nodes in their phylogenetic tree due to ‘large sampling errors’ [25]. A more recent phylogeny showing the independent origin of parthenogenesis for different species of Naryciinae using sequence data from mitochondrial DNA was produced by Grapputo et al. [26]. This study included several parthenogenetic *D. triquetrella* populations from Scandinavia, Switzerland and Austria, but only one sexual population (from Austria). Nevertheless, their results are in agreement with the allozyme study, also pointing at multiple origins of parthenogenesis.

In this study, we present the first phylogenetic analysis of multiple sexual and parthenogenetic *D. triquetrella* populations from a wide geographical range. We chose to use the mitochondrial genes COI and COII because they are easily sequenced, maternally inherited and relatively variable, making them ideal target genes to test for monophyly. Furthermore, differences in ploidy level and potential variation in crossing-over rates between sexuals and parthenogens are not expected to influence mutation patterns, in contrast to nuclear genes. We discuss the implications of our results in terms of the evolutionary history of parthenogenesis in this species, and whether or not the hypotheses initially posited by Seiler are plausible.

## Methods

### Species

*Dahlica triquetrella* (= *Solenobia triquetrella*) is a small (about 1 cm) moth within the family Psychidae, commonly known as bagworm moths. Males are winged but females are always wingless. Larvae construct cases (bags) composed of silk and small particles, which they inhabit until adult emergence. Due to its size, shape (triangular) and particle type, the cases of *D. triquetrella* can easily be distinguished from other *Dahlica* species [27] (but see discussion on *D. seileri*). Shortly after winter (upon snow melt), larvae climb upwards onto trees, walls and rocks where they pupate inside the cases. Emerging females either lay their eggs immediately inside the case (parthenogens) or sit on the outside of the case to attract males using pheromones (sexuals). Up to 100 eggs are produced, which hatch after several weeks. Whereas males can fly short distances, females are sessile. Other dispersal modes are unknown, but may include neonate larval ballooning as observed in other Psychidae [28] and occasional secondary transport, for example during spring flooding.

Three types of *D. triquetrella* are currently known: sexual diploids, parthenogenetic diploids and parthenogenetic tetraploids. Parthenogenetic and sexual diploids are only known from alpine and pre-alpine regions in Switzerland, Austria and Southern Germany [17,29], whereas tetraploid parthenogens occur throughout Europe [16] and are also found in North America [30]. Bag worm cases attributed to *D. triquetrella* from Baltic amber from the Eocene, 34–56 millions years old [31], indicate that genetic variation could potentially be large in this species due to its age.

Mechanistically, parthenogenesis in *D. triquetrella* is automictic with central fusion, where the two central polar nuclei fuse to restore the diploid or tetraploid status [3]: p. 67]. Because female Lepidoptera are generally achiasmatic [32], meiotic recombination is believed to be absent, leading to the maintenance of heterozygosity. However, unexplained genetic diversity in the closely related *D. fennicella*[33,34] could indicate that some form of recombination still occurs.

### Sample collection

**Figure 1:**
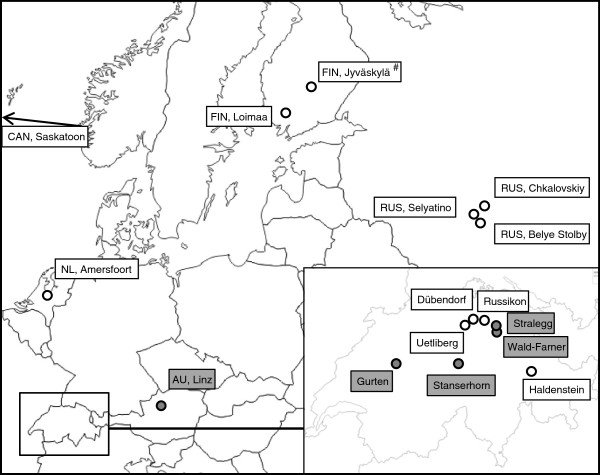
**Sampling locations where *Dahlica triquetrella *individuals were collected.** Sites where sexual individuals were found are indicated in grey. ^#^ Confirmed tetraploid population [35].

The major collection efforts were focused on locating sexual populations within alpine and pre-alpine areas of Switzerland and Austria (Figure 1). We specifically included some locations from which sexuals were previously found [17]. In addition, samples were obtained from several locations in Europe from which parthenogenetic types are known, and from Canada (Figure 1, Table 1).

**Table 1:** **Sampling sites, reproductive mode, and the number of *Dahlica triquetrella *individuals sequenced**

**Continent**	**Country**	**Location**	**Code**	**Latitude**	**Longitude**	**Reproductive mode**	**Females**	**Males**
Europe	Switzerland	Dübendorf	CH-2, CH-3	47.405 N	8.608 E	parthenogenetic	6	
		Haldenstein	CH-1 (334)^$^	46.874 N	9.521 E	parthenogenetic	1	
		Uetliberg	CH-8 (168)	47.343 N	8.489 E	parthenogenetic	5	
		Stanserhorn	CH-4 (189)	46.929 N	8.340 E	sexual	1	2
		Gurten	CH-5 (58)	46.921 N	7.437 E	sexual	2	4
		Wald-Farner	CH-6	47.286 N	8.952 E	sexual		1
		Strahlegg	CH-7 (216)	47.329 N	8.959 E	sexual	2	1
	Austria	Linz	AU	48.340 N	14.318 E	sexual		5
	Netherlands	Amersfoort	NL-1, NL-2	52.182 N	5.410 E	parthenogenetic	2	
	Finland	Jyväskylä*	FIN-2	62.2 N	25.7 E	parthenogenetic^#^	8	
		Loimaa	FIN-1	60.774N	23.007 E	parthenogenetic	4	
	Russia	Selyatino	RUS-2, RUS-3	55.510 N	36.946 E	parthenogenetic	2	
		Belye Stolby	RUS-4	55.323 N	37.847 E	parthenogeneti	1	
		Chkalovskaja	RUS-1	55.899 N	38.059 E	parthenogenetic	1	
North America	Canada	Saskatoon	CAN	52.1 N	106.7 W	parthenogenetic	2	

* Samples came from several sites within 25 km of these coordinates. ^$^Numbers between brackets refer to original sites from Seiler’s investigations (see [17]). ^#^Confirmed tetraploid population [35].

Larvae were hand-collected in late winter and early spring by inspecting trees, walls and rocks. Individual larvae were kept in separate vials until adult emergence. The reproductive mode was established at adult emergence; males and non-ovipositing females were considered sexual, and females producing eggs that later hatched were considered asexual. Adults were stored in 99% ethanol before DNA extraction. For one parthenogenetic population from Finland (FIN-2), tetraploid status was confirmed through flow-cytometry [35].

### Laboratory procedures

DNA was extracted from whole adults using the DNeasy Blood & Tissue Kit (QIAGEN) and eluted in 100μl of AE buffer. Amplification of an approximately 700 bp fragment of the Cytochrome Oxidase I was obtained using the universal forward primer LCO and the reverse primer HCO [36]. Amplification of an approx. 400 bp fragment of the COII was obtained using a forward primer specifically designed for Naryciinae (COII-M1-F: TTGGATTTAAACCCCATYTA) and the universal reverse primer C2-N-3389 [37]. Both primers included a M13 tail to allow the use of a standard fluorescent primer in the sequencing reaction [38]. Amplifications (20 μl total reaction volumes) were performed in a C1000 Thermo Cycler (Bio-Rad) using 10μL Premix B (Epicentre), 1 μl of each forward and reverse primer (10 μM), 0.5 μl of Failsafe Enzyme Mix (Epicentre), and 2 μl of DNA extraction. PCR conditions were: 95°C for 3min, followed by 30 cycles of 95°C for 30s, 50°C for 30s and 72°C for 1.5 min, followed by a final extension at 72°C for 5min. Amplifications were done in two rounds using 1 μl of the product of the first round for the second round. All 20 μl of the final PCR product was run on a 1% agarose gel with 10μM SYBRsafe DNA gel stain (Invitrogen). The band of the target size was cut and centrifuged through a 300 μl Finntip Filter (Thermo Labsystems) at 6000 rpm for 15 min for each sample.

Sequencing reactions were performed using 3.75μl BigDye Sequencing buffer (Applied Biosystems), 0.5 μl Ready Reaction Premix (Applied Biosystems), 13.75μl H_2_O, 1 μl of either the forward or reverse primer (3.2 μM), and 1 μl of the target band product. Cycling conditions were: 1 min at 96°C followed by 24 cycles of 10 s at 96°C, 5 s at 50°C, and 4 min at 60°C. The sequencing products were purified using an ethanol/EDTA/sodium acetate precipitation according to the BigDye® Terminator v3.1 Cycle Sequencing Kit manual (Applied Biosystems). Final products were analysed on an ABI 3130XL sequencer (Applied Biosystems). Sequences were checked and aligned using Seqscape 2.6 (Applied Biosystems). Final sequences were deposited in Genbank (accession numbers KC305201-KC305225).

### Data analysis

The number of haplotypes and variation in sequences were calculated for each gene using MEGA5. A haplotype network from the concatenated sequences was created with TCS 1.21 [39] to visualize the variation.

Our phylogenetic analyses included sequences of all encountered *D. triquetrella* haplotypes plus additional sequences from seven other Naryciinae species (five sexual and one parthenogenetic Dahliciini species from Finland [40] and *Narycia duplicella* from Belgium). First, we looked for the best model of nucleotide substitution for the concatenated sequences and for each gene separately with jModelTest [41]. Both the Akaike information criterion (AIC and corrected AIC) and the Bayesian Information Criterion (BIC) suggested the same best models (TIM2+G, weight=0.28) for the concatenated sequences, but various other models were included in the 95% confidence set of models, including parameter rich models (e.g. the General Time Reversible model GTR [42]) with a proportion of invariable sites (I) and/or a Gamma shaped rate variation (G). Similar results were obtained for COI (the Transition Model TIM2+I [43], weight=0.21) and COII (the Hasegawa-Kishino-Yano HKY model +G [44], weight=0.12–0.19) separately. Therefore, in the final analyses we implemented the most parameter rich model GTR+G+I.

A Maximum Likelihood (ML) phylogenetic tree was obtained for the concatenated sequences in PhyML 3.0 [44]. Eight substitution rate categories were used, with a neighbour-joining tree as start tree and tree topology was searched using Nearest Neighbor Interchange (NNI) and Subtree Pruning and Regrafting (SPR). The branch support was estimated from 500 bootstraps. A Bayesian phylogenetic tree was obtained with partitioning of the two genes using MrBayes 3.2 [45]. Four independent runs starting with random trees each with four MCMC chains (three hot and one cold) were run for four million generations (when stationarity had been reached, i.e. average standard deviation of split frequencies < 0.003) with trees sampled every 100 generations. The first one million generations (10000 trees in each run) were discarded (‘burn-in’ period) and the posterior probabilities were estimated for the remaining generations.

To specifically test whether the parthenogenetic haplotypes could be monophyletic, in contrast to the expectation that parthenogenesis evolved multiple times in *D. triquetrella*, we used the stepping-stone sampling approach in MrBayes 3.2 [46] on a dataset containing only the *D. triquetrella* haplotypes. We contrasted two constraints on topology: one with all parthenogenetic haplotypes being monophyletic and one where monophyly of parthenogens was not allowed.

## Results

**Figure 2:**
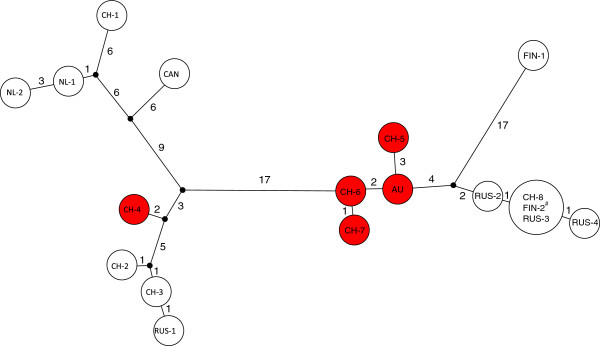
**Haplotype network for COI and COII in *Dahlica triquetrella.*** Haplotype network for the concatenated sequences of COI (658 bp) and COII (331 bp) sequences showing the number of variable sites. Haplotypes from sexual individuals are indicated in red. See Table 1 and Figure 3 for the geographic origin of each haplotype. ^#^ Confirmed tetraploid population [35].

We found sexual individuals from five locations in Switzerland and Austria. Parthenogenetic *D. triquetrella* were found from all other locations. Overall, we obtained COI and COII sequences from 50 individuals, with a total of 16 different haplotypes. Variation in COI (658 bp) was quite large (60 variable bases, 12 non-synonymous) with a maximum of 3.04% bp differences between sexuals and 5.32% between parthenogens. In COII (331 bp) variation was limited (25 variable bases, 1 non-synonymous) with a maximum of 1.51% bp differences between sexuals and 2.72% between parthenogens, which also was reflected in the reduced number of COII haplotypes. Most locations had unique sequences and either one or two similar ones; only one haplotype was found from multiple, but widely dispersed locations (Figure 2).

**Figure 3:**
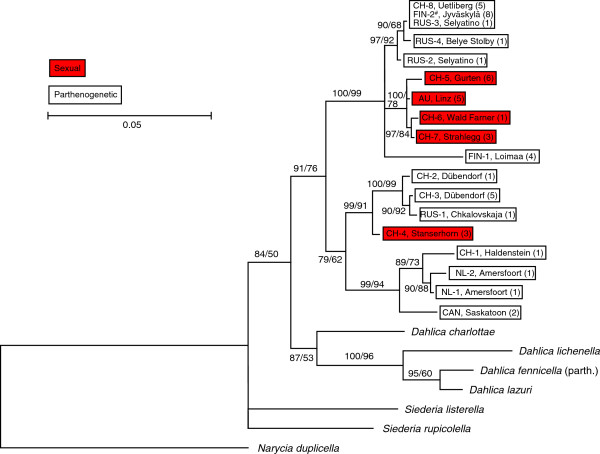
**Phylogenetic concensus tree of mtDNA from *Dahlica triquetrella.*** Post-burnin majority rule phylogenetic concensus tree of COI and COII concatenated sequences of sexual and parthenogenetic haplotypes and other Naryciinae species based on a GTR + I + G model of nucleotide substitution. Two branch support values are indicated. Left, the posterior probabilities obtained from a Bayesian analysis with partitioned sequences (four runs, each with 30.000 trees). Right, the likelihood values obtained from a Maximum Likelihood analysis with 500 bootstraps. For each haplotype, its geographic origin (see Table 1), the number of sequenced individuals, and the reproductive mode (red is sexual, white is parthenogenetic) are indicated. Note that *D. fennicella* is known only as parthenogenetic.^#^ Confirmed tetraploid population [35].

The ML and Bayesian analyses resulted in the same tree topology (Figure 3). Three main clades could be observed in *D. triquetrella.* However, the basal branches were not well supported, prohibiting conclusions on the phylogenetic relationships between the three clades and between *D. triquetrella* and closely related species. Specifically, our analyses show that the sexual haplotypes are paraphyletic, with one haplotype placed in a separate position from the other four and, consequently, that the parthenogens are not monophyletic. There was no strong regional separation of haplotypes, with Swiss and Russian genotypes widely distributed over the tree.

The stepping-stone sampling showed that the marginal log-likelihood of the constrained topology (all parthenogenetic haplotypes are monophyletic) was 85 natural log units larger than the unconstrained topology (−2213 vs. –2128), additionally suggesting that monophyly of parthenogens is highly unlikely (P <0.001, Bayes factor test).

## Discussion

The most salient finding of our study is that parthenogenetic lines of *D. triquetrella* are not monophyletic and must derive from different sexual ancestors. Specifically, three closely related sexual haplotypes, two from Switzerland and one from Russia, must have a different sexual ancestor than other haplotypes. This is in strong agreement with the original hypothesis that parthenogenesis evolved multiple times in this species. However, we did not find a clear ancestral sexual lineage to all haplotypes. This could have several causes. First, only few sexual populations were included, and thus existing sexual ancestors of the basal parthenogenetic lines may have been omitted. Secondly, the ancestral lines may have gone extinct after the appearance of the parthenogens. Seiler [17] stressed that sexual populations in the Alps would soon go extinct based on their small population sizes and the short-term disadvantages of sex. This also may explain why we observe so few clear sexual-parthenogenetic sister groups. Only a more exhaustive sampling of sexual populations could, perhaps, indicate which scenario is more likely. Alternatively, our results could indicate that sexuals have evolved from parthenogenetic ancestors. In Hymenoptera this may occur when e.g. *Wolbachia* infections are cured (e.g. [47]). In parthenogenetic *Dahlica*, *Wolbachia* is not believed to be the cause of parthenogenesis, as tests performed on *D. fennicella* did not detect *Wolbachia*[48]. A second possibility to retain sexuality is if parthenogenetic females would cross with males. Indeed, under laboratory conditions, diploid parthenogens have been observed to mate, leading to a portion of their offspring being male and female sexuals [20]. Also, fertilisation has been observed in tetraploid parthenogens under laboratory conditions, but due to the unequal chromosome numbers this cannot lead to fertile sexual offspring [20]. More data, in particular on ploidy levels and on variation in nuclear genes would be necessary to show potential reversals to sexuality in *D. triquetrella*.

Intra- and interlineage mtDNA divergence is often used to estimate when the transition to parthenogenesis occurred e.g. [11,12]. However, a common problem of estimating the age of parthenogenetic lineages is that we do not know if more closely related sexual lineages were not sampled or went extinct [11]. This is also the case in our study, and therefore, we cannot reliably estimate the number of independent transitions to parthenogenesis, nor estimate when they occurred. However, the current tree topology with many closely related parthenogenetic haplotypes on at least three major branches suggests that several parthenogenetic lineages have diversified and may have existed for a relatively long time. If we take the generally accepted mutation rate of 2.3% per million year for the COI gene [49] and consider the Dutch (NL-1, NL-2) and Swiss (CH-1) haplotypes as a diversified parthenogenetic lineage, the 1.4% divergence between NL-1 and CH-1 would suggest a minimum age of about 0.5 million years. If the Canadian haplotype is included, the minimum age of this lineage increases to approx. 1 million years. These values do not correspond with the idea that all parthenogenetic *D. triquetrella* evolved from sexuals in alpine refugia during the last glacial period (110 000–20 000 years ago) as previously proposed by Seiler [17]. Much more likely, the evolutionary processes in this species have been affected by the repeated contraction and expansion of ice sheets and glacial refugia over several glacial periods (see e.g. [50]).

Almost every sampling site yielded a different haplotype, including the sexual sites. This is in agreement with the earlier allozyme study [22] that showed that almost every sexual and parthenogenetic site in Switzerland had unique alleles. This may reflect the fact that populations in the Alps already existed and have been isolated from each other for a long time. Lokki et al. [22] also found two main genotypes in Finland, an eastern and a western type, suggesting two colonisation routes. They suggested that outside the Alps only few (ancient) parthenogenetic lines had spread, whereas within the Alps, new parthenogenetic lines were forming all the time. Although our data potentially includes the two genotypes from Finland, we also found that a large variety of haplotypes are present outside the Alps. In many cases, these are closely related to Swiss parthenogenetic samples, refuting the idea of Lokki et al. [22] that tetraploid parthenogenetic lineages outside the Alps are older than those found inside the alpine region. One parthenogenetic haplotype was found over a wide geographic area (Russia, Finland and Switzerland) suggesting that it has more recently spread. This further indicates that the distribution of parthenogenetic types cannot be explained by one invasion of a few haplotypes from the Alps immediately after the last glacial period. Interestingly, evidence was recently found for spruce and pine surviving in refugia in Northern Scandinavia during the last glacial period [51]. It is imaginable that *D. triquetrella,* which is found frequently in spruce forests, may also have survived there, or in other refugia outside the Alps.

The variation in mtDNA within *D. triquetrella* is often larger than that observed between several other Naryciinae species, and exceeds many proposed values to delimit species [52]. The large variation in mtDNA may be correlated with observed variation in morphology [53], and also could explain why there are several questionable species and subspecies described that are very locally distributed [16]. For instance, *D. seileri*, which differs mainly in larval case size [54], is now considered just another tetraploid form of *D. triquetrella*[30]. Only careful phylogenetic and morphometric analyses can reveal whether separate species status will hold.

Considering the high number of parthenogenetic haplotypes, the likelihood that several parthenogenetic lineages have diversified, and that these lineages are geographically widespread shows that parthenogenesis in *D. triquetrella* is very advantageous in this species. One of many hypotheses (see e.g. [55,56]) is that the winglessness of the females may play a role. Sexual females cannot actively look for mates and may become mate-limited, especially in sparse populations [40,57]. Further, in poorly dispersing species, parthenogens should have a very clear advantage in colonisation of new areas [7]. Indeed, all psychid parthenogenetic species are wingless and parthenogenesis is relatively more common in insect groups with many wingless species [8]. Secondly, recombination, generating new genotypes, may still occur in the parthenogens, as suggested from genetic studies in the closely related partenogenetic *D. fennicella*[33,34]*.*

To better understand the evolution of the transition to parthenogenesis in *D. triquetrella* additional data would be needed. First, as mentioned before, only few sexual sites were sampled in our study. Seiler and colleagues sampled about 60 other sites in Switzerland e.g. [17] and a few populations are also known from Austria and Southern Germany [23,29]. Local extinction (e.g. due to deforestation and urbanisation of sites; pers. obs. LNSS), remoteness, snow in late winter and the short period of emergence made it particularly challenging to collect. Further, since only maternally inherited mtDNA was analysed, our results cannot give an indication if hybridisation has played a role in this species as suggested by Tomiuk and Loeschke [25]. Analyses of nuclear gene sequences with appropriate levels of variation would be necessary. An important aspect of the evolution of parthenogenesis in *D. triquetrella* is the ploidy of the parthenogens. In this study, tetraploidy was confirmed for only one of the Finnish (FIN-2) populations [35]. Seiler [17] indicates that individuals coming from populations corresponding to our CH-1 and CH-8 were tetraploid, but we have no information on the other parthenogenetic samples. It thus remains to be seen if diploid parthenogens could be ancestral to tetraploids, or if they form a separate lineage. Finally, the phylogenetic relation between the Z/ZZ and ZW/ZZ sexual and parthenogenetic types remains to be investigated [22].

## Conclusions

In conlusion, our study clearly shows that different parthenogenetic lineages are present in *D. triquetrella* with a large divergence in mtDNA. This strongly suggests multiple transitions to parthenogenesis (corroborating previous hypotheses), and that some parthenogenetic lineages have evolved much earlier than the last ice age (in contrast to previous hypotheses). *Dahlica triquetrella* is thus another species where parthenogenesis has been a successful adaptation and has been present for a relatively long time.

## Competing interests

The authors declare that they have no competing interests.

## Authors’ contributions

JAE carried out sampling and laboratory analyses, performed the sequence alignment and analyses, and drafted the manuscript. JJ participated in its design and coordination, and helped to draft the manuscript. LNSS carried out sampling and laboratory analyses, and helped to draft the manuscript. All authors read and approved the final manuscript.

## Authors’ information

JAE is an evolutionary ecologist working mainly on insect interactions and has been working on Naryciinae since 2007. JJ is an evolutionary ecologist working on host-parasite interactions and ecological genetics of natural populations. LNSS is an evolutionary ecologist with particular interests in genetic responses to environmental change.
